# Factors associated with general practitioners' routines and comfortability with assessing female genital cutting: a cross-sectional survey

**DOI:** 10.1186/s12913-023-09085-4

**Published:** 2023-01-25

**Authors:** Mai Mahgoub Ziyada, R. Elise B Johansen, Mona Berthelsen, Inger-Lise Lien, Bothild Bendiksen

**Affiliations:** 1 Norwegian Centre for Violence and Traumatic Stress Studies, PB: 181 Nydalen, 0409 Oslo, Norway; 2 Institute of Health and Society, Faculty of Medicine, University of Oslo, PB: 1130 Blindern, 0318 Oslo, Norway

**Keywords:** Female Genital Mutilation/cutting, Healthcare, General practitioners, Management, Training, Knowledge, Competence

## Abstract

**Background:**

Female genital cutting (FGC) may cause a series of health problems that require specialized healthcare. General practitioners (GPs) are gatekeepers to specialized healthcare services in Norway. To refer girls and women subjected to FGC to appropriate services, GPs need to assess whether the health problems reported by these patients are related to FGC. However, we do not know to what degree GPs assess FGC as a potential cause of the patients' health problems. We also know little about the GPs' patterns of training and knowledge of FGC and their effect on the GPs' assessment of FGC as a potential cause of health problems.

**Method:**

We employed a cross-sectional online survey among GPs in Norway to examine: 1) patterns of received training on FGC, self-assessed knowledge, and experiences with patients with FGC-related problems and 2) the association between these three factors and the GPs' assessment of FGC as a potential cause of patients' health problems. A total of 222 GPs completed the survey. Data were analysed using binary logistic regression, where we also adjusted for sociodemographic characteristics.

**Results:**

Two-third of the participants had received training on FGC, but only over half received training on FGC-related health problems. Over 75% of the participants stated a need for more knowledge of FGC typology and Norwegian legislation. While the majority of the participants assessed their knowledge of FGC medical codes as inadequate, this was not the case for knowledge of the cultural aspects of FGC. Female GPs were more likely to have experience with patients with FGC-related health problems than male GPs. Among GPs with experience, 46% linked health problems to FGC in patients unaware of the connection between FGC and such health problems. GPs were more likely to assess FGC as a potential cause of health problems when they had experience with patients having FGC-related problems and when they assessed their knowledge of FGC typology and FGC-related medical codes as adequate.

**Conclusion:**

To improve their assessment of FGC as a potential cause of patients' health problems, GPs should receive comprehensive training on FGC, with particular emphasis on typology, health problems, and medical codes.

**Supplementary Information:**

The online version contains supplementary material available at 10.1186/s12913-023-09085-4.

## Background

Female Genital Cutting (FGC) refers to all traditional practices involving injuring the female external genitalia for non-medical reasons [1]. The World Health Organization (WHO) differentiates between four types of FGC that vary in severity, with type III (infibulation) being the most extensive form. Girls and women with FGC, type III, in particular, have a higher risk of experiencing a series of short- and long-term health problems than those who are not cut [2, 3]. Long-term health problems include genitourinary, obstetrical, and sexual health problems, such as cysts, keloids, dysmenorrhea, hematometra, urinary tract infections, poor urinary flow, perineal tears, prolonged labor, dyspareunia, reduced sexual desire and satisfaction, and symptoms of anxiety, and depression [2–12].

Although FGC is primarily prevalent in 31 countries in Africa, Asia, and the Middle East, where more than 200 million girls and women have been subjected to FGC [13], it has also spread to other parts of the world. For example, in Europe, it is estimated that more than half a million immigrant girls and women have been subjected to FGC [14], of whom 17,000 live in Norway [15]. Many of these girls and women could need specialized healthcare. For example, those subjected to the most extensive form often require surgical intervention (deinfibulation) to facilitate sexual intercourse and childbirth [16, 17]. Moreover, recent evidence indicates that girls and women subjected to FGC, who live in a context where the majority condemn FGC, such as Western countries, are more likely to report psychosexual problems than those in countries of origin [10, 18–22]. Consequently, many Western countries have established FGC specialized clinics to meet their affected immigrant population's potential healthcare needs [23]. These countries have also developed training modules and practical and clinical guidelines to help healthcare providers deal with FGC-related health problems and prevent and avert FGC [17, 23–31].

Since 2000, Norway has had six action plans with earmarked funding to address FGC [32–36]. Despite the predominant focus on preventive and protective measures, most action plans have also emphasized providing healthcare for those with FGC. Therefore, in 2004, as a national healthcare offer on FGC, several women’s outpatient departments started to provide specialized services for affected girls and women [23].

In Norway, general practitioners (GPs) are typically the first contact between patients and the healthcare system [37]. GPs make primary diagnoses, treat problems that do not require specialized healthcare, prescribe drugs, issue sick leaves, and assess whether the patients need a referral to specialized healthcare services. Upon referral from GPs, the hospitals' outpatient departments provide specialized healthcare services to the patients. To lower the threshold for patients with FGC-related health problems to access specialized healthcare, some hospitals allow women with FGC to contact the women's outpatient departments for appointments directly and thus forego referral from their GPs. However, some girls and women with FGC-related health problems do not benefit from self-referral. They either lack knowledge of the FGC-specialized healthcare services or are unaware of or uncertain whether there is a connection between these problems and FGC [38]. The GPs will continue to play a major role in helping these patients access the appropriate specialized services. Therefore, GPs need to proactively assess whether relevant health problems experienced by these patients are related to FGC. This proactive assessment would most likely entail that GPs take the initiative to ask women from FGC practicing countries about their FGC status when they present with health problems potentially related to FGC. They also need to feel comfortable to discuss FGC with their patients and consequently assess FGC thoroughly as a potential cause.

We do not know, neither in Norway nor other western countries, whether GPs assess FGC as a potential cause of a patient's health problems when such assessment is relevant. However, qualitative studies indicate a mutual silence on FGC during GPs' consultations [38–40]. Therefore, we need to know more about factors that can improve GPs' practices in assessing FGC among relevant patient groups presenting with potential FGC-related health problems.

Many studies in Western countries have investigated healthcare providers' knowledge, attitudes, and practices concerning FGC [41–59]. Most studies [41–44, 46–48, 52–59] have included nurses, midwives, gynecologists, and obstetricians, followed by pediatricians [46–49, 51, 55]. Only three studies [45–47] included GPs. Furthermore, most studies have assessed whether healthcare providers have received training on FGC, their general knowledge of FGC (types, affected groups, reasons for the practice, health complications, and legislation), attitudes towards medicalization, experience with women subjected to FGC, and relevant practices (educating patients, reporting cases of FGC to child protection services, and performing deinfibulation). Almost all studies have concluded that healthcare providers' knowledge of FGC was insufficient for important FGC-related practices such as clinical management and prevention.

Nevertheless, only a few studies [46, 47, 50, 51, 53, 54, 60] have examined the association between the received training on FGC and the healthcare providers' knowledge or performance of certain FGC-related practices (e.g., identification of FGC cases and notification to child protection authorities). We identified only one [60] study that has investigated care provision. This latter study found a positive association between the healthcare providers' confidence in providing FGC-related healthcare and knowledge of health complications, experience with women with FGC, and more than five years of clinical experience. Still, none of the studies has assessed the association between received training and acquired knowledge and the GPs' routines and patterns of assessing FGC as a potential cause of relevant health problems. This article aims to fill some of the knowledge gaps.

Our main objectives are to examine: a) patterns of received training on FGC among GPs in Norway, their self-assessed knowledge, and their experiences with patients with FGC-related problems; and b) the association between these three factors and the GPs' assessment of FGC as a potential cause of patients' health problems. Our null hypotheis is that there is no statistically significant association between GPs' training levels, self-assessed knowledge, and experiences with patients with FGC-related health problems and the GPs' assessment of FGC as a potential cause of patients' health problems.

## Methods

We conducted a cross-sectional anonymous online survey between June and July 2019.

### Study population, recruitment, and participants

The study population was all registered GPs in Norway in 2019, i.e., 4774 GPs [61]. We engaged an external company (IQVIA Institute for Human Data Science) to help recruit study participants. IQVIA had an e-mail list of 4100 GPs in Norway who had not reserved themselves against being contacted by the company. IQVIA sent a request to participate accompanied by information about the study and a link to the online survey to these 4100 GPs. IQVIA also sent one reminder. Additionally, we e-mailed the same information to chief medical officers in all Norwegian municipalities requesting further distribution to local GPs. We also published this information on the Journal of the Norwegian Medical Association, three relevant websites, and relevant Facebook groups.

Out of the 4100 e-mail addresses administered by IQVIA, 306 were no longer valid. Hence, only 3794 GPs received a request to participate. In total, 223 completed the questionnaire, which constitutes a response rate of 5.8%. Unfortunately, one respondent had missing data on all sociodemographic variables and was excluded from the analyses.

### Measurement

We designed an online self-administered questionnaire (Additional file 1) built on insight from an extensive literature review, our research questions, and expert opinions. We then piloted the questionnaire among seven GPs for clarity, adequacy, and relevance of questions and response alternatives. The pilot resulted in minor adjustments to some questions and response alternatives.

#### Explanatory variables

Explanatory variables included:

- a) Sociodemographic variables (gender, age, length of experience, location of practice, and country of basic medical training);
- b) Received training on FGC during basic medical studies (hereafter undergraduate), after completion of medical studies including specialization and continuous medical training (hereafter post-graduate), and on FGC-related health problems;
- c) Self-assessed knowledge of FGC (cultural aspects of FGC, WHO classification, Norwegian legislation, and FGC-related medical codes derived from the Norwegian adaptation of the International Classification of Diseases codes ICD-10);
- d) Experience with patients with FGC-related health problems.

We recoded response alternatives of some variables that included more than two response alternatives into dichotomous ones. For example, the response alternatives of yes, partly, and no for the variables "received training on FGC and self-assessed knowledge of FGC" were dichotomized to yes (yes and partly).

#### Outcome variables

We assumed that to proactively assess whether FGC is the underlying cause of health problems experienced by women originating from FGC-practicing countries, GPs need to know the women's FGC status and feel comfortable talking to them about FGC. Accordingly, we used the following two outcome variables as outcome indicators for the GP's assessment of FGC as a potential cause of patients' health problems: feeling discomfort talking to patients about FGC and routinely asking about FGC in consultations with patients with potential FGC-related obstetrical, urogenital, mental and sexual health problems. The response alternatives of yes, somewhat, and no for the variable "feeling discomfort talking to patients about FGC" were dichotomized to yes (yes and somewhat) and no. Similarly, yes, sometimes, and no response alternatives were dichotomized to yes (yes and sometimes) and no for routinely asking about FGC in consultations with patients from FGC-practicing countries presenting with pregnancy, urogenital, sexual, or mental health problems.

### Statistical analysis

We conducted descriptive analyses for the explanatory and outcome variables and presented the results using frequency and percent. To identify potential confounders of demographic characteristics, we compared each category of the explanatory variables by each demographic variable using the Chi-square test and binary logistic regression. After that, binary logistic regression analyses were performed to examine the association between our outcome and explanatory variables, adjust for possible confounders, and look for interactions between the explanatory variables. Missing data were excluded from these analyses. Results from the binary logistic regression analyses were summarized using crude odds ratio (OR), adjusted odds ratio (aOR), p-value, and confidence interval (CI). Results with a *p*-value < 0.05 were considered statistically significant. All tests were two-tailed. Analyses were conducted using IBM SPSS statistics version 26.

### Ethics

The Norwegian Regional Committee for Medical and Health Research Ethics (REK) approved the study. Requests to participate encompassed background information, the purpose of the study, and that the study was anonymous and no personally identifiable information about the respondents was stored. Subsequent filling and submission of the survey were considered as informed consent.

## Results

### Sociodemographic characteristics

Our sample consisted of more female (54.5%, *n* = 121) than male (45.5%, *n* = 101) participants. More than half of the participants were in the age group 30–49 years (58.1%, *n* = 129) and had 6–25 years of experience after basic medical training (56.8%, *n* = 126). The majority (72.1%, *n* = 160) had undertaken their basic medical training partly or fully in Norway. See Table 1 for the distribution of all explanatory and outcome variables.

**Table 1:** Distribution of all explanatory and outcome variables

Variable		*N* = 222	Percent
		n	(%)
Gender	Female	121	54,5
	Male	101	45,5
Age (years)	< 30	2	0,9
	30–39	61	27,5
	40–49	68	30,6
	50–59	41	18,5
	≥ 60	50	22,5
Location of practice	Urban	131	59,0
	Rural	91	41,0
Length of experience (years)	≤ 5	20	9,0
	6–15	81	36,5
	16–25	45	20,3
	26–35	51	23,0
	≥ 36	25	11,3
Country of medical training	Norway	160	72,1
	Abroad	62	27,9
Any undergraduate training on FGC	Yes	103	46.4
	No	117	52.7
	No response	2	0.9
Any post-graduate training on FGC	Yes	92	41.4
	No	129	58.1
	No response	1	0.5
Training on FGC related health problems	Yes	117	52.7
	No	104	46.8
	No response	1	0.5
Adequate knowledge of FGC medical codes (ICD-10 or NCMP-NCPS-NCPR)	Yes	50	22.5
	No	170	76.6
	No response	2	0.9
Adequate knowledge of the cultural aspects of FGC	Yes	185	83.3
	No	35	15.8
	No response	2	0.9
Need for more knowledge of WHO FGC-typology	Yes	177	79.7
	No	43	19.4
	No response	2	0.9
Need for more knowledge of FGC-related legislation in Norway	Yes	173	77.9
	No	47	21.2
	No response	2	0.9
Experience with patients with FGC-related health problems	Yes	77	34.7
	No	145	65.3
	No response	0	0
Feeling discomfort talking to patients about FGC	Yes	42	18.9
	Partly	74	33.3
	No	104	46.8
	No response	2	0.9
Ask about FGC with urogenital problems	Yes	92	41.4
	Sometimes	71	32.0
	No	53	23.9
	No response	6	2.7
Ask about FGC with pregnancy	Yes	105	47.3
	Sometimes	60	27.0
	No	52	23.4
	No response	5	2.3
Ask about FGC with mental health problems	Yes	36	16.2
	Sometimes	79	35.6
	No	99	44.6
	No response	8	3.6
Ask about FGC with sexual health problems	Yes	97	43.7
	Sometimes	74	33.3
	No	46	20.7
	No response	5	2.3

### Received training, self-assessed knowledge, and experience with FGC-related health problems

Almost two-thirds of the participants (68%, *n* = 151) received training on FGC as part of either their undergraduate or post-graduate training (Data not shown in table). However, just over half of the participants (53%, *n* = 117) received training on FGC-related health problems (Table 1). After adjusting for other sociodemographic variables, the likelihood of receiving undergraduate training on FGC was significantly higher among those who received their basic training in Norway than those who had studied abroad. In contrast, this likelihood significantly decreased with increased length of experience since completion of basic medical training (Table 2).

**Table 2:** Relationship between sociodemographic characteristics and other explanatory variables

	Received training						Adequate knowledge				Need for more knowledge				Experience with patients with FGC-related health problems	
	Undergraduate (*n* = 220)		post-graduate (*n* = 221)		On FGC-related health problems (*n* = 221)		FGC medical codes (*n* = 220)		Cultural aspects (*n* = 220)		WHO typology (*n* = 220)		Legislation (*n* = 220)		(*n* = 222)	
	OR (CI)	aOR (CI)	OR (CI)	aOR (CI)	OR (CI)	aOR (CI)	OR (CI)	aOR (CI)	OR (CI)	aOR (CI)	OR (CI)	aOR (CI)	OR (CI)	aOR (CI)	OR (CI)	aOR (CI)
	P	P	P	P	P	P	P	P	P	P	P	P	P	P	P	P
Gender	2.25 (2.30 -3.87)	1.63 (0.85–3.14)	1.36 (0.79–2.33)	1.59 (0.90–2.81)	1.61 (0.94–2.74)	1.45 (0.83–2.52)	1.20 (0.63 -2.27)	1.06 (0.54–2.07)	2.01 (0.96–4.20)	2.33 (1.08–5.03)	1.68 (0.86–3.29)	1.65 (0.82–3.33)	1.85 (0.96–3.55)	1.82 (0.92–3.60)	2.29 (1.29–4.08)	2.36 (1.29–4.30)
	0.004^*^	0.143	0.268	0.114	0.081	0.188	0.577	0.872	0.063	0.032^*^	0.131	0.162	0.065	0.087	0.005^*^	0.005^*^
Age	0.42 (0.32–0.56)	0.84 (0.46–1.57)	1.27 (1.00–1.61)	1.20 (0.69–2.07)	0.87 (0.68–1.09)	1.06 (0.62- 1.81)	0.77 (0.58–1.03)	0.47 (0.24–0.93)	1.13 (0.82–1.57)	0.66 (0.33–1.36)	0.84 (0.63–1.13)	1.40 (0.69–2.80)	0.84 (0.63–1.11)	1.46 (0.74–2.88)	0.95 (0.74–1.21)	0.94 (0.53–1.67)
	0.000^*^	0.591	0.050	0.519	0.224	0.832	0.075	0.030^*^	0.452	0.260	0.246	0.351	0.223	0.279	0.675	0.833
Length of experience	0.40 (0.30–0.53)	0.36 (0.19–0.69)	1.25 (1.00–1.58)	1.13 (0.67–1.91)	0.85 (0.67–1.06)	0.80 (0.48–1.33)	0.88 (0.67–1.16)	1.65 (0.87–3.12)	1.27 (0.92–1.75)	1.95 (0.98–3.89)	0.79 (0.59–1.05)	0.63 (0.33–1.23)	0.78 (0.59–1.02)	0.60 (0.32–1.15)	0.97 (0.77–1.23)	1.12 (0.65–1.92)
	0.000^*^	0.002^*^	0.053	0.640	0.142	0.392	0.355	0.129	0.146	0.059	0.099	0.180	0.073	0.126	0.806	0.696
Location of practice	0.69 (0.40–1.19)	0.62 (0.32–1.21)	1.53 (0.88–2.66)	1.46 (0.83–2.57)	1.32 (0.77–2.25)	1.29 (0.75–2.24)	0.78 (0.42–1.48)	0.78 (0.41–1.49)	1.24 (0.60–2.56)	1.17 (0.55–2.46)	0.63 (0.31–1.27)	0.63 (0.31–1.28)	0.60 (0.30–1.18)	0.58 (0.29–1.17)	1.74 (0.98–3.10)	1.74 (0.96–3.15)
	0.182	0.159	0.130	0.185	0.318	0.361	0.449	0.452	0.569	0.683	0.193	0.200	0.140	0.131	0.061	0.068
Country of medical training	2.57 (1.38–4.79)	6.99 (3.13–15.59)	0.98 (0.54–1.78)	0.80 (0.43–1.49)	1.41 (0.78–2.54)	1.48 (0.80–2.74)	1.32 (0.64–2.73)	1.53 (0.72–3.25)	1.20 (0.55–2.64)	1.05 (0.46–2.39)	0.73 (0.34–1.59)	0.77 (0.34–1.73)	0.84 (0.41–1.76)	0.90 (0.42–1.94)	0.78 (0.43–1.44)	0.69 (0.36–1.32)
	0.003^*^	0.000^*^	0.954	0.481	0.252	0.211	0.456	0.273	0.642	0.911	0.425	0.528	0.649	0.783	0.433	0.261

*Note: n total number included in the analysis. Gender is coded 0 (males), 1 (females). Categories of age and length of experience were recoded into continuous variables. Age: coded 1 (*< *30 years) to 5 (*≥ *60 years), median* = *3 (40 – 49 years). Length of experience* = *years of practice after basic medical training: coded 1 (*≤ *5 years) to 5 (*≥ *36 years of practice after basic medical training), median* = *3 (16 – 25 years of practice). Location of practice codes 0 (rural), 1 (urban). Country of medical training: coded 0 (abroad), 1 (Norway). All other explanatory variables are coded 0 (no) and 1 (yes). Statistical significance level at α* ≤ *0.05, OR crude odds ratio, CI confidence interval, aOR adjusted odds ratio*

Most participants (83%, *n* = 185) assessed their knowledge to be adequate on the cultural aspects of FGC, while only 23% (*n* = 50) assessed their knowledge to be adequate on medical codes for FGC. Almost 80% (*n* = 177) of the participants stated a need for more knowledge of the WHO typology and about 78% (*n* = 173) on FGC legislation (Table 1). After adjusting for other sociodemographic variables, female participants were more likely to assess their knowledge of the cultural aspects of FGC to be adequate than their male counterparts. Further, the likelihood for the GPs to assess their knowledge of ICD-10 medical codes on FGC as adequate significantly decreased with increased age (Table 2).

Just over one-third of the participants (35%, *n* = 77) reported having experience with patients with health problems related to FGC (Table 1). Among those with experience, about 68% (*n* = 52) were females, 39% (*n* = 30) in the age group 30–49, 68% (*n* = 52) had their practice in an urban setting, and 69% (*n* = 53) had their medical training fully or partly from Norway. Among those with experience, just less than half (46%, *n* = 35) had identified cases of FGC-related health problems among patients who were unaware of the link between these problems and FGC (Data not shown in table).

The likelihood of having experience with patients with FGC-related health problems was only significantly associated with gender, with female participants more likely to have experience than male participants (Table 2).

### Feeling discomfort talking to patients about FGC

Feeling discomfort talking to patients about FGC is the first of the two outcome variables that we used to examine the GPs' assessment of FGC as a potential cause of patients' health problems. More than half of the participants (52%, *n* = 116) reported feeling some level of discomfort talking to patients about FGC (Table 1).

Table 3 shows the association between feeling discomfort talking to patients about FGC and the explanatory variables. There was no significant association between sociodemographic characteristics nor received training and GPs feeling discomfort talking to patients about FGC. Before adjusting for other explanatory variables, the likelihood of feeling discomfort talking to patients about FGC was significantly higher among those who reported a need for more knowledge of the WHO typology and FGC legislation than those who did not. In contrast, the likelihood of feeling discomfort talking to patients about FGC was significantly lower among those who self-assessed their knowledge of both FGC medical codes and cultural aspects of FGC to be adequate, and those with experience with patients with FGC-related problems than their counterparts. After adjusting for other explanatory variables, the likelihood of feeling discomfort talking to patients about FGC was only significantly higher among those who reported a need for more knowledge of the WHO typology than those who did not. On the other hand, the likelihood of feeling discomfort talking to patients about FGC was only significantly lower among those with knowledge of FGC medical codes than those without (Table 3).

**Table 3:** Association between feeling uncomfortable talking to patients about FGC and the explanatory variables

Variable	Feeling discomfort talking to patients about FGC (*n* = 220)			
	OR (CI)	P	aOR (CI)	P
**Gender**	0.98 (0.58–1.67)	0.941	0.91 (0.49–1.68)	0.758
**Age**	0.89 (0.70–1.12)	0.311	0.78 (0.44–1.39)	0.400
**Length of experience**	0.89 (0.71–1.12)	0.309	1.22 (0.69–2.14)	0.498
**Any undergraduate training on FGC**	1.09 (0.64–1.86)	0.746	1.39 (0.67–2.92)	0.378
**Any post-graduate training on FGC**	0.63 (0.36–1.08)	0.089	0.77 (0.38–1.57)	0.470
**Training on FGC- related health problems**	0.64 (0.38–1.10)	0.106	0.83 (0.37–1.83)	0.636
**Knowledge of FGC medical codes (ICD-10 or NCMP-NCPS-NCPR)**	0.41 (0.22–0.79)	0.008^*^	0.39 (0.19–0.79)	0.009^*^
**Adequate knowledge of the cultural aspects of FGC**	0.45 (0.21–0.98)	0.044^*^	0.64 (0.27–1.51)	0.310
**Need for more knowledge of WHO typology**	3.21 (1.57–6.57)	0.001^*^	2.36 (1.04–5.34)	0.040^*^
**Need for more knowledge of FGC-related legislation**	2.99 (1.51–5.93)	0.002^*^	1.99 (0.90–4.38)	0.089
**Experience with patients with FGC-related health problems**	0.64 (0.37–1.11)	0.114	0.71 (0.38–1.33)	0.288

*Note: n total number included in the analysis. Gender is coded 0 (males), 1 (females). Categories of age and length of experience were recoded into continuous variables. Age: coded 1 (*< *30 years) to 5 (*≥ *60 years), median* = *3 (40 – 49 years). Length of experience* = *years of practice after basic medical training: coded 1 (*≤ *5 years) to 5 (*≥ *36 years of practice basic medical training), median* = *3 (16 – 25 years of practice). Location of practice codes 0 (rural), 1 (urban). Country of medical training: coded 0 (abroad), 1 (Norway). All other explanatory variables are coded 0 (no) and 1 (yes). Statistical significance level at α* ≤ *0.05, OR crude odds ratio, CI confidence interval, aOR adjusted odds ratio*

### Asking about FGC in consultations with patients with potential FGC-related health problems

Over three-quarters of the participants (77%, *n* = 171) asked sometimes or routinely about FGC in their consultations with patients from FGC-practicing countries presenting with sexual problems. Furthermore, nearly three-quarters of the participants asked sometimes or routinely about FGC in their consultations with patients from FGC-practicing countries presenting either during pregnancy (74.3%, *n* = 165) or with urogenital problems (73.4%, *n* = 163) compared to merely over half of the participants (51.8%, *n* = 115) in the case of mental health problems (Table 1). Table 4 shows the association between the participants' routines of asking about FGC in consultations with patients with potential FGC-related health problems and our explanatory variables.

**Table 4:** Associations between routine medical inquiries concerning potential FGC-related health problems and all explanatory variables

Variable	Ask about FGC in consultations with patients from FGC practicing countries presenting with															
	**Urogenital problems (*n* = 216)**				**Pregnant patients (*n* = 217)**				**Mental health problems (*n* = 214)**				**Sexual health problems (*n* = 217)**			
	**OR (CI)**	**p**	**aOR (CI)**	**p**	**OR (CI)**	**p**	**aOR (CI)**	**p**	**OR (CI)**	**p**	**aOR (CI)**	**p**	**OR (CI)**	**p**	**aOR (CI)**	**p**
**Gender**	2.30 (1.22, 4.33)	0.010^*^	1.79 (0.83–3.86)	0.136	2.98 (1.55, 5.72)	0.001^*^	2.14 (0.97–4.75)	0.061	1.22 (0.71, 2.10)	0.464	0.05 (0.01–0.38)	0.004^*^	1.79 (0.93, 3.45)	0.083	1.31 (0.58–2.93)	0.515
**Age**	0.96 (0.73, 1.26)	0.746	1.79 (0.87–3.69)	0.114	0.98 (0.74, 1.29)	0.863	1.63 (0.78–3.42)	0.194	1.49 (1.16, 1.91)	0.002^*^	1.39 (0.72–2.71)	0.329	0.96 (0.72, 1.27)	0.751	1.65 (0.79–3.44)	0.183
Gender ^*^Age											2.75 (1.48–5.11)	0.001^*^				
**Length of experience**	0.89 (0.69–1.16)	0.393	0.55 (0.27–1.12)	0.100	0.95 (0.73–1.23)	0.687	0.75 (0.36–1.56)	0.444	1.37 (1.08–1.74)	0.009^*^	0.81 (0.45–1.48)	0.498	0.90 (0.69–1.19)	0.456	0.61 (0.29–1.26)	0.183
**Any undergraduate training on FGC**	1.86 (0.98–3.53)	0.058	1.31 (0.52–3.30)	0.568	2.10 (1.09, 4.05)	0.027^*^	1.61 (0.62–4.15)	0.328	1.05 (0.61, 1.81)	0.849	1.31 (0.59–2.90)	0.505	1.69 (0.86–3.30)	0.127	1.24 (0.48–3.20)	0.655
**Any post-graduate training on FGC**	3.46 (1.66, 7.19)	0.001^*^	3.22 (1.21–8.54)	0.019^*^	2.59 (1.29, 5.21)	0.008^*^	1.50 (0.57–3.92)	0.413	1.89 (1.08, 3.31)	0.025^*^	1.24 (0.58–2.68)	0.579	2.35 (1.14, 4.84)	0.021^*^	1.68 (0.64–4.40)	0.291
**Training on FGC- related health problems**	2.76 (1.44, 5.28)	0.002^*^	0.80 (0.30–2.16)	0.665	3.34 (1.72, 6.51)	< 0.001^*^	1.69 (0.62–4.65)	0.305	1.78 (1.03, 3.06)	0.039^*^	1.08 (0.46–2.55)	0.862	2.55 (1.30, 5.03)	0.007^*^	1.10 (0.41–3.00)	0.849
**Knowledge of FGC medical codes**	4.82 (1.64, 14.11)	0.004^*^	5.08 (1.62–15.87)	0.005^*^	2.79 (1.11, 6.98)	0.029^*^	2.47 (0.90–6.82)	0.081	3.02 (1.49, 6.09)	0.002^*^	3.84 (1.72–8.58)	0.001^*^	5.43 (1.61, 18,36)	0.006^*^	5.52 (1.55–19.66)	0.008^*^
**Adequate knowledge of the cultural aspects of FGC**	2.82 (1.32, 6.03)	0.007^*^	1.88 (0.77–4.60)	0.164	2.53 (1.18, 5.43)	0.018^*^	1.44 (0.57–3.64)	0.440	1.94 (0.93, 4.06)	0.078	1.35 (0.57–3.22)	0.496	2.27 (1.03, 5.01)	0.042^*^	1.43 (0.57–3.61)	0.443
**Need for more knowledge of WHO typology**	1.07 (0.50, 1.62)	0.859	1.15 (0.43–3.08)	0.780	1.73 (.83, 3.59)	0.143	2.72 (0.98–7.53)	0.054	1.01 (0.52, 1.98)	0.971	0.99 (0.42–2.32)	0.977	1.16 (.52, 2.58)	0.713	1.58 (0.57–4.40)	0.384
**Need for more knowledge of legislation**	0.72 (0.32, 1.62)	0.428	0.74 (0.26–2.05)	0.556	0.63 (0.27, 1.45)	0.278	0.36 (0.11–1.18)	0.090	0.91 (0.47, 1.77)	0.783	1.21 (0.51–2.86)	0.666	0.51 (0.20, 1.29)	0.153	0.45 (0.14–1.47)	0.186
**Experience with patients with FGC-related health problems**	3.41 (1.56, 7.46)	0.002^*^	2.87 (1.22–6.74)	0.016^*^	9.52 (3.28, 27.63)	< 0.001^*^	7.90 (2.52–24.81)	< 0.001^*^	2.28 (1.27, 4.09)	0.006^*^	2.24 (1.13–4.46)	0.021^*^	5.96 (2.55, 15.84)	< 0.001^*^	5.34 (1.91–14.91)	0.001^*^

*Note: n total number included in the analysis. Gender is coded 0 (males), 1 (females). Categories of age and length of experience were recoded into continuous variables. Age: coded 1 (*< *30 years) to 5 (*≥ *60 years), median* = *3 (40 – 49 years). Length of experience* = *years of practice after basic medical training: coded 1 (*≤ *5 years) to 5 (*≥ *36 years of practice after basic medical training), median* = *3 (16 – 25 years of practice). Location of practice codes 0 (rural), 1 (urban). Country of medical training: coded 0 (abroad), 1 (Norway). All other explanatory variables are coded 0 (no) and 1 (yes). Statistical significance level at α* ≤ *0.05, OR crude odds ratio, CI confidence interval, aOR adjusted odds ratio*

Before adjusting for other explanatory variables, the female gender was significantly associated with an increased likelihood of asking about FGC in consultation with patients from FGC-practicing countries presenting during pregnancy or with urogenital problems. Increased length of experience and age were significantly associated with an increased likelihood of asking about FGC when patients from FGC- practicing countries present with mental health problems (Table 4). Participants with post-graduate training on FGC, training on FGC health problems, self-assessed adequate knowledge of FGC medical codes and cultural aspects of FGC, and experiences with patients with FGC-related health problems were more likely than their counterparts to ask about FGC in consultations with patients from FGC practicing countries presenting during pregnancy or with urogenital and sexual health problems. The likelihood of asking about FGC in consultations with pregnant patients from FGC-practicing countries was also significantly higher among participants who had any undergraduate training on FGC than their counterparts. In consultations with patients from FGC-practicing countries presenting with mental health problems, participants who had any post-graduate training on FGC, training on FGC health problems, self-assessed adequate knowledge of FGC medical codes, and experience with patients with FGC-related health problems were significantly more likely to ask about FGC than those who had not (Table 4).

After adjusting for other explanatory variables, gender was only significantly associated with the likelihood of asking about FGC in consultations with patients from FGC-practicing countries presenting with mental problems, where female participants had a lower chance of asking about FGC than their male counterparts. Receiving post-graduate training on FGC was only significantly associated with asking about FGC in consultation with patients from FGC-practicing countries presenting with urogenital problems. Those who received the training were more likely to ask about FGC than those who did not. Participants with knowledge of FGC medical codes were significantly more likely to ask about FGC in their consultations with patients from FGC-practicing countries presenting with urogenital, mental, and sexual health problems than those without knowledge of medical codes. Finally, participants with experience with FGC-related health problems were significantly more likely to ask about FGC in consultations with patients from FGC-practicing countries presenting during pregnancy or with urogenital, sexual, and mental health problems.

## Discussion

GPs have a key role in the healthcare of girls and women subjected to FGC. This article first examined patterns of received training on FGC, self-assessed knowledge, and experiences with FGC-related health problems among 222 GPs in Norway. After that, it examined the associations between received training, self-assessed knowledge of FGC, and experiences with patients with FGC-related health problems and two outcome variables that we used as indicators for the GPs' assessment of FGC as a potential cause of patients' health problems. These two outcome variables were "feeling discomfort talking to patients about FGC" and "routines of asking about FGC in consultations with patients from FGC-practicing countries presenting during pregnancy or with urogenital, sexual, and mental health problems."

Almost two-thirds of our participants received training on FGC during either undergraduate or post-graduate training, which is a higher proportion than those reported elsewhere [46, 51, 53, 54, 57]. In Norway, three out of five medical schools provide training on FGC as part of their undergraduate training [62]. Nevertheless, training on FGC varies in content and duration (between 45–120 min). Considering the many aspects of FGC that need to be covered within such a timeframe, it is not surprising that just over half of the participants received training on FGC-related health problems. Similar to a French study [45], we found that female and younger GPs were more likely to report receiving training on FGC than male and older participants. This latter finding could reflect females' specific sensitization/interest in the topic [45, 47] and a relatively recent introduction of FGC into the medical curricula [62].

While most participants assessed their knowledge of the cultural aspects of FGC as adequate, they did not consider their knowledge adequate when it came to the medical codes. Further, over three-quarters of the participants expressed a need for more knowledge of FGC legislation and the WHO typology on FGC. Our findings are consistent with other studies on knowledge of typology [42, 46, 47, 63], legislation [42, 45, 52, 56, 63], and medical codes [51], but not cultural aspects [48, 52]. It is also possible that the cultural aspects of FGC are particularly emphasized in the training curricula on FGC in Norway. It is also possible that GPs in our study overestimated their knowledge of the cultural aspects of FGC. Previous studies [46, 47] indicate that healthcare providers tend to overestimate their knowledge of FGC. We did not assess the GPs' actual knowledge versus their self-assessed knowledge. Hence, the GPs' levels of actual knowledge of all aspects of FGC in Norway could be lower than those reported in this article. Regardless, our findings indicate that GPs in Norway need comprehensive FGC training, emphasizing typology, health problems, medical codes, and legislation.

In addition, we found female GPs to be more likely to have experience with patients with FGC-related health problems than male GPs, which might be related to a previous finding showing that some women with FGC prefer female healthcare providers [39, 52, 64]. Among the GPs who had experiences with patients with FGC-related health problems, 46% experienced that patients were unaware of the connection between their health problems and FGC. While we do not know the accuracy level of the GPs' diagnoses of health problems as FGC-related, this finding still indicates that the assessment of whether FGC causes the patients' health problems should not be left entirely to the patients. In addition, recent qualitative articles [38, 39] have revealed that some women subjected to FGC prefer their GPs to take the initiative to ask about their FGC status.

Feeling discomfort talking to patients about FGC could negatively affect the GPs' consultations with women subjected to FGC and the assessment of whether the women's health problems are due to FGC [65]. We found that feeling discomfort talking to patients about FGC was significantly higher among GPs who reported a need for more knowledge of the WHO typology than those who did not. In contrast, feeling discomfort talking to patients about FGC was significantly lower among those with knowledge of FGC medical codes than among those without such knowledge. Further, we found that the GPs with adequate knowledge of FGC medical codes were significantly more likely to ask about FGC in their consultations with patients from FGC-practicing countries presenting with urogenital, mental, and sexual health problems than their counterparts. These findings highlight the importance of clinical knowledge of FGC for the assessment of whether FGC is a cause of obstetrical, urogenital, sexual, and mental health problems experienced by girls and women subjected to FGC. We agree with Johnsdotter and Essén [66] that having a too strong focus on FGC as the presumed cause of such symptoms may lead to misdiagnosis. Nevertheless, failing to assess whether FGC is an underlying cause of symptoms could also lead to misdiagnosis and suffering. Careful consideration of whether FGC is an underlying cause for health problems, whenever relevant, is necessary to provide quality care for these women.

### Implications for clinical practice and future training

In Norway, permitting self-referral to the FGC-specialized healthcare service for women with FGC is a commendable initiative. Nevertheless, self-referral would not benefit girls and women with FGC-related health problems who do not link these problems to FGC or are unaware of the FGC-specialized clinics. GPs will continue to play a significant role in these patients' access to appropriate specialized services. Further, assessing whether FGC causes health problems requires both medical knowledge and diagnostic competency. Hence, such an assessment should not be left entirely to the women. We recommend that Norwegian plans of action on FGC recognize and emphasize the key role that GPs play in the clinical management of patients with FGC-related health problems. Further, GPs should be provided with comprehensive training on FGC at all levels of training (undergraduate, graduate, and continuous medical training). While this comprehensive training on FGC should probably continue to provide knowledge of cultural aspects, there is a critical need to emphasize typology, health problems, medical codes, and legislation.

## Strengths and limitations

The study's low response rate challenges the external validity and generalisability of our findings. To recruit participants, we depended on an available list of valid e-mail addresses of GPs administered by IQVIA, constituting about 79.5% of all GPs in Norway. Selection bias regarding who accepted and reserved against inclusion in IQVIA's e-mail list is possible. Still, it is reasonable to assume that such decisions are unrelated to the aims of the current study. It is also possible that selection bias regarding experience with patients with FGC-related health problems influenced the participation in the study (i.e., those with experience were more likely to participate).

However, we recruited a substantial group (65%) of participants who reported no experience. Gender and age distribution of our participants were close to that of the target population [61]. Thus, even though we would be cautious about generalizing our findings to all GPs in Norway, we consider the knowledge produced by the current study to be a valuable addition to the research field and an important contribution to informing the Norwegian decision-makers and healthcare providers. To our knowledge, this is the first study to use statistical analyses to explore the association between GPs' training and self-assessed knowledge of FGC and their comfort and routines regarding the assessment of FGC as a potential cause of patients' health problems.

## Conclusion

Our findings emphasize that GPs have a key role in the clinical management of patients with FGC-related health problems, particularly patients unaware of the connection between their health problems and FGC. Furthermore, we found that GPs were more likely to assess FGC as a potential cause of health problems when they had experience with patients having FGC-related problems and knowledge of FGC typology and medical codes. Therefore, to help GPs proactively assess FGC as a potential cause of patients' health problems, they should receive comprehensive training on FGC with emphasis on typology, health problems, and medical codes.

Finally, future research could benefit from adopting a qualitative approach that builds on our findings to provide a deeper and more insightful understanding of GPs experiences with the assessment with potential FGC-related health problems in Norway beyond the factors explored in this article.

## Supplementary Information

**Additional file 1.**

## Data Availability

All data generated or analysed during this study are included in this published article and its supplementary information files.

## Abbreviations

aOR: Adjusted odds ratio
CI: Confidence interval
EU: European Union
FGC: Female genital cutting
GP: General practitioner
ICD: International classification of diseases
OR: Odd ratio
REK: The Norwegian Regional Committee for Medical and Health Research Ethics
WHO: World Health Organization
