# Supplementary material for: Factors associated with general practitioners' routines and comfortability with assessing female genital cutting: a cross-sectional survey
Source: BMC Health Serv Res. 2023 Jan 25;23:82. doi: 10.1186/s12913-023-09085-4 (PMC9878807; doi:10.1186/s12913-023-09085-4)
Supplement: Supplementary file 1 — Additional file 1. [file 12913_2023_9085_MOESM1_ESM.pdf]

Physical and psychological healthcare for women subjected to female genital mutilation: a study among general practitioners in Norway

1. Background information

1.1. Gender

☐ Female

☐ Male

1.2. How old are you?

☐ < 30 years

☐ 30-39 years

☐ 40-49 years

☐ 50-59 years

☐ ≥ 60 years

1.3. In which health region do you work?

☐ Norway – region North

☐ Norway – region Middle

☐ Norway – region West

☐ Norway – region South

☐ Norway – region East

1.4. Do you work in:

☐ City

☐ Rural area

1.5. How many years of work experience do you have after graduating from medical school?

☐ ≤ 5 years

☐ 6-15 years

☐ 16-25 years

☐ 26-35 years

☐ ≥ 36 years

1.6. How many years of work experience do you have after being certified as specialist in general practice?

☐ I am not yet certified as specialist

☐ ≤ 5 years

☐ 6-15 years

☐ 16-25 years

☐ 26-35 years

☐ ≥ 36 years

1.7. Did you receive your medical degree from Norway?

☐ Yes

☐ Partly

☐ No

1.8. Did you receive training on female genital mutilation (in general) during your medical studies?

☐ Yes

☐ Partly

☐ No

1.9. After graduating from medical school, have you received training on female genital mutilation (in general) during your further/continuous medical training?

☐ Yes

☐ Partly

☐ No

1.10. Have you received training on health complications related to female genital mutilation?

☐ Yes

☐ Partly

☐ No

2. Knowledge and competence on female genital mutilation

Classification and legislation

2.1. Do you feel you need more knowledge on the WHO classification of the different types of female genital mutilation (I – IV)?

☐ Yes

☐ Partly

☐ No

2.2. Are you familiar with the medical codes for female genital mutilation (ICD-10, or NCMP-NCPS-NCPR)?

☐ Yes

☐ Partly

☐ No

2.3. Do you feel you need to know more about the legislation related to female genital mutilation?

☐ Yes

☐ Partly

☐ No

Cultural competence

2.4. Do you think you have adequate knowledge concerning why female genital mutilation is practiced?

☐ Yes

☐ Partly

☐ No

2.5. Do you think you have adequate knowledge concerning the traditional and cultural meaning of female genital mutilation for the women?

☐ Yes

☐ Partly

☐ No

2.6. Do you feel uncomfortable to talk to affected patients about female genital mutilation?

☐ Yes

☐ Partly

☐ No

2.6.1. If you answered yes in question 2.6., is this because of patient-related issues?

☐ Yes

☐ Partly

☐ No

2.6.2. If you answered yes in question 2.6., is this because of issues related to you?

☐ Yes

☐ Partly

☐ No

2.7. In your consultations with female patients originating from countries where female genital mutilation is practiced (See the map), do you routinely ask the patients whether they have been subjected to female genital mutilation when/if they present with any of the following health related issues?

2.7.1. Urogenital problems

☐ Yes

☐ Sometimes

☐ No

2.7.2. Pregnancy

☐ Yes

☐ Sometimes

☐ No

2.7.3. Mental health problems

☐ Yes

☐ Sometimes

☐ No

2.7.4. Sexual health problems

☐ Yes

☐ Sometimes

☐ No

2.8. Have you ever been consulted by women who have health problems related to female genital mutilation?

☐ Yes

☐ No

3. Consultations

This section is only visible for those who select the "yes" option in question 2.8.

3.1. Have you ever been consulted by women who directly indicated that their health problems are related to female genital mutilation?

☐ Yes

☐ No

3.2. Have you ever been consulted by women who have health problems related to female genital mutilation, but they were unaware of the link between their problems and female genital mutilation?

☐ Yes

☐ No
